# High-stimulation-rate ABR predicts persistent auditory pathway dysfunction in neonates with severe hyperbilirubinemia: a 6-month longitudinal study

**DOI:** 10.3389/fneur.2025.1687189

**Published:** 2026-01-12

**Authors:** Yuanquan Li, Qingchun Pan, Xingya Li, Qianhui Pei, Bei Li, Renli Huang

**Affiliations:** 1 Affiliated Hospital of North Sichuan Medical College, Nanchong, Sichuan, China; 2 Longquanyi District Maternal and Child Health Hospital, Chengdu, China; 3 Longchang People’s Hospital, Sichuan, Longchang, China

**Keywords:** auditory brainstem response, high stimulation rate, hyperbilirubinemia, longitudinal study, neonates

## Abstract

**Objective:**

This study aims to analyze high-stimulation-rate auditory brainstem response (ABR) outcomes in neonates diagnosed with hyperbilirubinemia (NH) and assess its clinical relevance.

**Methods:**

This study utilized a prospective longitudinal cohort design. It included ABR tests conducted at 11.1 and 51.1 stimulations per second, alongside serum total bilirubin (TSB) measurements for 28 neonates (56 ears) with severe NH, 28 neonates (56 ears) with mild-to-moderate NH, and 28 neonates (56 ears) from a healthy control group (CG) during their hospital stay. We compared the differences among these groups. Additionally, ABR tests at both high and low stimulation rates were administered to neonates with severe and mild-to-moderate NH at ages 3 and 6 months to compare both inter-group and intra-group variations.

**Results:**

(1) During hospitalization, interpeak latency (IPL) differences between waves I and V (ΔIPL I-V) at high (51.1 stimulations per second) and low (11.1 stimulations per second) stimulation rates showed that the severe NH group exhibited longer ΔIPL I-V compared to the healthy CG (*p* < 0.05) and the mild-to-moderate NH group (*p* < 0.05). The mild-to-moderate group also demonstrated longer ΔIPL I-V compared to the CG (*p* < 0.05). Correlation analysis between TSB and ΔIPL I-V in neonates with NH during hospitalization revealed no significant correlation in the CG (*r* = 0.203, *p* = 0.300) and the mild-to-moderate group (*r* = 0.116, *p* = 0.558), but a positive correlation in the severe group (*r* = 0.712, *p* < 0.05). (2) At 3 months, the ΔIPL I-V in the severe group was notably longer than that in the mild-to-moderate group (*p* < 0.05). This pattern persisted at 6 months (*p* < 0.05). (3) Within-group comparisons showed that in the severe group, ΔIPL I-V was shorter at both 3 and 6 months than during hospitalization (*p* < 0.05), with no significant changes between these two ages (*p* > 0.05). In the mild-to-moderate group, ΔIPL I-V remained unchanged across all examined periods (*p* > 0.05).

**Conclusion:**

High-stimulation-rate ABR is a potent tool for detecting auditory deficits in neonates with NH. Neonates with severe NH exhibit persistent auditory abnormalities, whereas those with mild-to-moderate conditions display only transient alterations. These findings underscore the value of high-stimulation-rate ABR in the early identification of neonates at high risk, recommending its incorporation into routine follow-up protocols for those with severe NH.

## 1 Introduction

Neonatal hyperbilirubinemia (NH), commonly observed during the neonatal period, is defined by a total serum bilirubin (TSB) level exceeding 220.6 μmol/L (12.9 mg/dL) in full-term neonates. Globally, the incidence of NH ranges from 60 to 80% (1). Although interventions like phototherapy and exchange transfusion have significantly reduced the incidence of kernicterus, bilirubin neurotoxicity continues to cause damage to the auditory pathway, a serious complication that requires attention. Numerous studies have confirmed a strong link between high TSB levels and neuronal toxicity (2). Current findings suggest that even subthreshold TSB levels for exchange transfusion can still cause selective impairment of the auditory brainstem pathway, potentially leading to subtle hearing deficits (3). Research shows that the prevalence of hearing impairment in severely jaundiced neonates is 10 to 50 times higher than that in their healthy peers (4). This dysfunction is characterized by a specific “post-cochlear lesion,” where cochlear hair cell function is preserved, but neural conduction is disrupted. This condition can lead to false-negative results in standard otoacoustic emission screenings, posing a significant challenge for early clinical diagnosis (5).

The auditory brainstem response (ABR) is an objective method used to evaluate the functionality of the auditory pathway from the auditory nerve to the brainstem, and is crucial for neonatal hearing screening and diagnosis (6). Prior research has shown that certain interpeak intervals and latencies in the ABR of normal-hearing neonates, recorded at a conventional low stimulation rate (11.1 times/s), are abnormal (7). However, when ABR is conducted at this rate, it primarily assesses the overall function of the auditory pathway with limited sensitivity to early synaptic transmission anomalies. Recent neuroelectrophysiological studies have indicated that high-stimulation-rate ABR (51.1 times/s) improves the detection of diminished neural conduction efficiency caused by conditions such as ischemia, hypoxia, or toxic damage, by highlighting synaptic fatigue (8). Earlier research emphasized the unique diagnostic value of high-stimulation-rate ABR for identifying central lesions, such as those caused by vertebrobasilar artery ischemia (9). These findings suggest new directions for studying auditory function in NH neonates. Nevertheless, current clinical research still faces three primary limitations: (1) the lack of characteristic high-stimulation-rate ABR spectra across various bilirubin concentration gradients; (2) an incomplete understanding of neurofunctional recovery patterns after bilirubin normalization; and (3) the absence of objective electrophysiological markers to measure the extent of damage.

This study introduces “the difference between interpeak intervals I-V at high and low stimulation rates (△IPLI-V)” as a novel biomarker for evaluating auditory pathway injury in neonates with NH. Using a prospective longitudinal design, our research systematically uncovers several pivotal findings for the first time: (1) the identification of a distinct abnormal ABR pattern at high stimulation rates in neonates diagnosed with severe NH; (2) a dose–response relationship between TSB peak levels and △IPLI-V; (3) the progressive pattern of auditory injury transitioning from transient functional impairment to permanent structural damage. These insights surpass the constraints of conventional ABR, which traditionally provides only qualitative data, by offering a quantifiable scale for injury assessment suitable for clinical application.

## 2 Materials and methods

### 2.1 Study design

This investigation was designed as a prospective longitudinal cohort study. All enrolled neonates with NH and healthy controls were recruited consecutively at hospitalization between January 2022 and January 2025. Baseline assessments were performed prior to the initiation of any treatment. Follow-up assessments were then prospectively conducted at 3 and 6 months of age for the NH groups.

### 2.2 Participants

This study enrolled a cohort of 56 NH neonates (112 ears) and 28 healthy neonates (56 ears) from the Longquanyi District Maternal and Child Health Hospital in Chengdu. The NH group comprised 28 neonates with severe NH (TSB > 320.6 μmol/L) and 28 with mild-to-moderate NH (TSB 220.6–320.6 μmol/L), diagnosed according to the criteria in the 4th edition of “Practical Neonatology” (10). The sample size was determined based on effect sizes from previous neonatal ABR studies, which indicated that 25–30 participants per group provides 80% power to detect clinically meaningful differences (effect size *f* > 0.4) at *α* = 0.05. A post-hoc power analysis confirmed 99% power for detecting our primary outcome (△IPL I-V difference between groups).

The inclusion criteria for NH patients include: (1) a birth weight exceeding 2,000 g, singleton status, a gestational age of more than 35 weeks, and the ability to complete all required tests; (2) admission within 7 days post-birth, followed by the completion of high- and low-stimulation-rate ABR, TEOAE, and acoustic impedance tests during hospitalization, and at three and 6 months of age. TEOAE must show passing results in both ears, and acoustic impedance must exhibit a type A tympanogram; (3) adherence to the diagnostic criteria for NH as outlined in the fourth edition of “Practical Neonatology”: patients are categorized into mild (220.6–270.6 μmol/L), moderate (270.6–320.6 μmol/L), and severe (>320.6 μmol/L) groups, based on TSB levels (10). The exclusion criteria include: (1) twin or multiple births; (2) the need for resuscitation due to asphyxia, as indicated by an Apgar score of 0–4 at 1 min or 0–6 at 5 min; (3) positive screening results for neonatal genetic metabolic diseases or glucose-6-phosphate dehydrogenase deficiency; (4) significant hypoxemia or the need for mechanical ventilation for more than 5 days during hospitalization; (5) a family history of permanent hearing impairment in childhood, or in utero infections caused by cytomegalovirus, rubella virus, herpes virus, syphilis, or Toxoplasma gondii; (6) craniofacial malformations, including those of the auricle and ear canal; (7) maternal use of ototoxic drugs during pregnancy; (8) bacterial meningitis; (9) syndromes or genetic diseases clinically evident or suspected to be related to hearing impairment; (10) unreliable or incomplete clinical data, or instances where parents refused treatment, opted for self-discharge, or the child died. The inclusion criteria for the healthy CG are as follows: (1) a birth weight greater than 2000 g, singleton status, a gestational age over 35 weeks, and normal postnatal assessment indicators; (2) TEOAE showing passing results during hospitalization, with a type A tympanogram; (3) the ability to cooperate with all tests. The exclusion criteria for the healthy CG include: (1) TEOAE failure in one or both ears during hospitalization; (2) lack of parental cooperation with the tests; (3) complications with other neonatal diseases. This study received approval from the Ethics Committee of the Affiliated Hospital of North Sichuan Medical College and the Maternal and Child Health Hospital of Longquanyi District, Chengdu City (Approval No.: 2020ER035-1). All participating patients or their guardians signed informed consent forms. The test flowchart is depicted in Supplementary Figures 1.

### 2.3 ABR protocol and procedures

All participants underwent otoscopy of the external auditory canal to clear any secretions. The Capella otoacoustic emissions analyzer was employed to assess otoacoustic emissions in both ears across frequencies of 0.5, 1, 2, and 4 kHz, documenting whether emissions were detected at these frequencies. The initial ABR testing for all neonates was performed within 24 h of admission, and crucially, before the initiation of any specific treatment for hyperbilirubinemia. Auditory evoked potentials were recorded using the Biologic system in an electromagnetically shielded room (11). The skin was cleansed with 95% alcohol. The recording electrode was positioned at the midpoint of the forehead along the hairline, reference electrodes were affixed to both mastoids, and the ground electrode was situated at the root of the eyebrow. The inter-electrode impedance was maintained at ≤ 6 kΩ, and ambient noise levels did not exceed 25 dB(A) (11). Test parameters included an amplifier band-pass filter set from 100 to 3,000 Hz, a gain of 100 k, and 2000 superimpositions. Sound was delivered using insert earphones with the stimulation being a short tone at an intensity of 80 dBnHL (11). Each participant was tested at stimulation rates of 11.1 and 51.1 times per second. The latencies of waves I, III, and V, as well as the intervals between waves I-III, III-V, and I-V, were recorded at each rate. The procedure was repeated thrice to calculate and compare variations in wave latencies and inter-wave intervals under the two stimulation rates. The testing conditions complied with the standards set forth in GB/T16403 (1996) (12). Electrode placement and impedance requirements followed the specifications of the Biologic evoked potential system manufacturer’s protocol (11).

### 2.4 Outcome measures

The primary outcome measure was the △IPL I-V. Secondary outcomes included the absolute latencies of waves I, III, and V, and the interpeak latencies for I-III, III-V, and I-V, each analyzed separately for the 11.1/s and 51.1/s stimulation rates.

### 2.5 Statistical analysis

Statistical analysis was conducted using SPSS software, version 26.0. Measurement data were reported as (x ± s). For comparisons between two groups, an independent samples *t*-test was employed, while an analysis of variance was utilized for comparisons among multiple groups. A repeated measures analysis of variance was applied to assess changes in each index before and after the intervention. Categorical data were presented as *n* (%), and the chi-square test was used to compare these data between groups. The Pearson test was conducted for correlation analysis. The significance level was set at *α* = 0.05. Bonferroni correction was applied for multiple comparisons, and a *p*-value of less than 0.05 was considered of statistical significance.

## 3 Results

### 3.1 General information

There were insignificant differences in gestational age, birth weight, body length, gender, or mode of delivery between the severe NH group, the mild-to-moderate NH group, and the healthy CG (*p* > 0.05). The peak TSB levels recorded for each group were as follows: for the severe NH group, 390.15 ± 32.78 μmol/L; for the mild-to-moderate NH group, 272.13 ± 21.83 μmol/L; and for the healthy CG, 189.39 ± 17.58 μmol/L. Significant differences in peak TSB levels were noted among these groups (*p* < 0.001), as detailed in Table 1.

**Table 1:** Comparison of general data among children in the severe NH group, the mild-to-moderate NH group, and the CG.

Groups	Severe NH group (*n* = 28 cases, 56 ears)	Mild-to-moderate group (*n* = 28 cases, 56 ears)	Healthy CG (*n* = 28 cases, 56 ears)	*x*^2^/F	*p*
Gestational age (weeks)	38.53 ± 1.62	38.00 ± 1.82	38.24 ± 1.39	0.75	0.48
Birth weight (g)	3.88 ± 0.59	4.03 ± 0.60	4.07 ± 0.70	0.73	0.49
Height (cm)	54.50 ± 3.43	55.42 ± 3.44	55.02 ± 3.99	0.46	0.64
Gender				0.68	0.71
Male (%)	14(50.00)	17(60.71)	16(57.14)		
The way of birth				0.67	0.72
Cesarean section (%)	12(42.86)	13(46.43)	15(53.57)		
The maximum value of TSB (μmol/L)	390.15 ± 32.78	272.13 ± 21.83*	189.39 ± 17.58*#	459.57	< 0.05

*p<0.05 vs. severe NH group; # *p*<0.05 vs. mild-to-moderate group.

### 3.2 Analysis of high-stimulation rate ABR results in children with severe NH, mild-to-moderate NH, and the CG during hospitalization

This section presents the results from ABR tests conducted at both high and low stimulation rates during the hospitalization of neonates diagnosed with NH, and a healthy CG. The details are provided in Table 2. (1) At a low stimulation rate of 11.1 times per second, no significant differences were observed in the latencies of waves I, III, and V, or in the inter-wave intervals among the groups with severe NH, mild-to-moderate NH, and the healthy controls (*p* > 0.05). (2) At a high stimulation rate of 51.1 times per second, the latencies of wave I, wave III, and wave V, as well as the inter-wave intervals between waves III and V, and I and V in the severe NH group, were significantly greater than those observed in the healthy CG (*p* < 0.05). Additionally, the difference in the inter-peak latency between waves I and V (△IPL I-V) at both high and low stimulation rates was significantly greater in the severe NH group compared to the healthy controls (*p* < 0.05). (3) At the same high stimulation rate of 51.1 times per second, the latencies of wave III and V, and the inter-wave interval between waves I and V in the severe NH group, were significantly greater than those in the mild-to-moderate NH group (*p* < 0.05). Furthermore, the △IPL I-V in the severe NH group exceeded that in the mild-to-moderate NH group (*p* < 0.05). (4) Continuing at this high stimulation rate, the latencies of wave I, wave III, and wave V, along with the inter-wave intervals between waves III and V, and I and V in the mild-to-moderate NH group, were significantly greater than those in the healthy CG (*p* < 0.05). The △IPL I-V in the mild-to-moderate NH group was also significantly greater than that in the healthy CG (*p* < 0.05).

**Table 2:** Comparison of high-stimulation rate ABR among severe NH group, mild-to-moderate NH group, and CG during hospitalization (ms).

Groups	Stimulation rate	Latentperiod			Interpeak latency			51.1 times per second and 11.1 times per second Inter-interval differences of waves I-V
		I	III	V	I-III	III-V	I-V	△IPL I-V
Severe NH group	11.1 times/s	1.72 ± 0.25	4.57 ± 0.20	6.66 ± 0.18	2.84 ± 0.29	2.10 ± 0.26	4.94 ± 0.32	–
Mild-to-moderate NH group	11.1 times/s	1.65 ± 0.23	4.53 ± 0.21	6.62 ± 0.19	2.89 ± 0.34	2.08 ± 0.29	4.97 ± 0.28	–
Healthy CG	11.1 times/s	1.65 ± 0.20	4.49 ± 0.21	6.60 ± 0.21	2.84 ± 0.29	2.11 ± 0.30	4.96 ± 0.29	–
	*F*	2.00	1.91	1.35	0.32	0.16	0.13	
	*p*	0.14	0.15	0.26	0.73	0.86	0.88	
Severe NH group	51.1 times/s	1.83 ± 0.25	4.8 ± 0.17	7.13 ± 0.18	2.97 ± 0.32	2.33 ± 0.25	5.30 ± 0.33	0.36 ± 0.42
Mild-to-moderate NH group	51.1 times/s	1.78 ± 0.21	4.67 ± 0.20^*^	6.93 ± 0.24^*^	2.89 ± 0.24	2.25 ± 0.31	5.14 ± 0.32^*^	0.17 ± 0.40^*^
Healthy CG	51.1 times/s	1.68 ± 0.18^*#^	4.6 ± 0.17^*#^	6.68 ± 0.20^*#^	2.92 ± 0.29	2.08 ± 0.28^*#^	5.01 ± 0.27^*#^	0.05 ± 0.41^*#^
	*F*	6.94	16.21	64.66	1.08	11.35	12.94	8.28
	*p*	0.05	<0.05	<0.05	0.34	<0.05	<0.05	<0.05

*p < 0.05 vs. severe NH group; #*p* < 0.05 vs. mild-to-moderate group.

**Figure 1:**
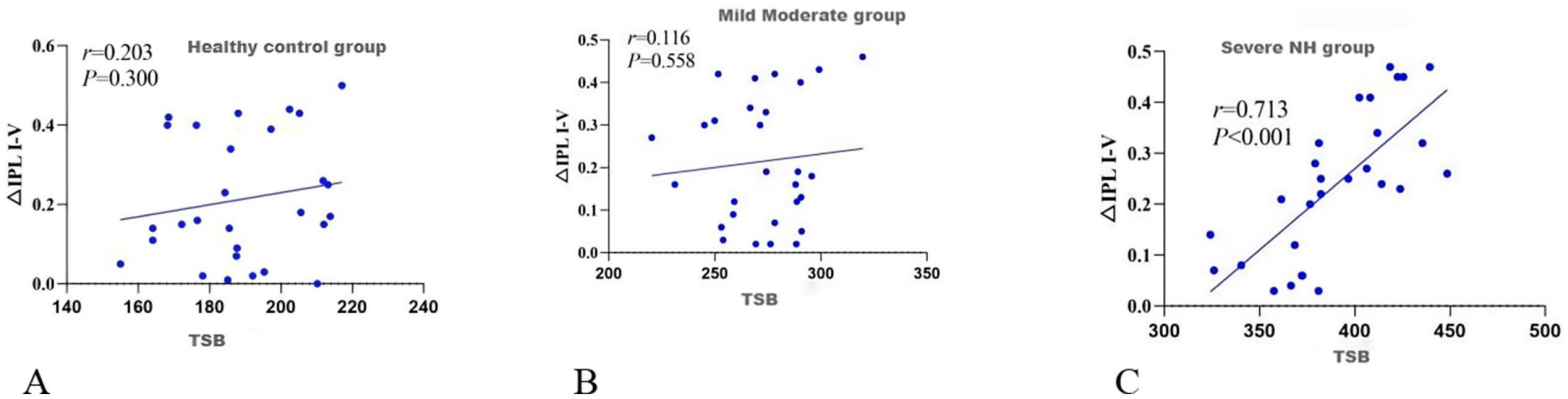
Correlation between peak TSB and ΔIPL I-V: **(A)** Correlation graph for the CG; **(B)** Correlation graph for mild-to-moderate NH; **(C)** Correlation graph for severe NH. TSB represents the peak value observed during hospitalization, and ΔIPL I-V indicates the change in the worst-affected ear.

The analysis of the correlation between the peak TSB levels during hospitalization and the differences in the interpeak latency of waves I-V (△IPL I-V) at stimulation rates of 51.1 times/s and 11.1 times/s in NH revealed distinct patterns. In the CG, there was no significant correlation between the peak TSB values and △IPL I-V (*r* = 0.203, 95% CI: −0.194-0.542, *p* = 0.300), as depicted in Figure 1A. Similarly, no significant correlation was observed in neonates with mild-to-moderate NH (*r* = 0.116, 95% CI: −0.278-0.476, *p* = 0.558), as illustrated in Figure 1B. Conversely, we identified a positive link in neonates with severe NH (*r* = 0.712, 95% CI: 0.523–0.836, *p* < 0.05), as shown in Figure 1C.

### 3.3 Inter-group comparison and analysis of ABR at high and low stimulation rates at 3 months of age

The comparison of ABR results at high and low stimulation rates between the severe NH group and the mild-to-moderate NH group at 3 months of age is presented in Table 3. At a low stimulation rate of 11.1 times/s, the latency measurements for waves I, III, and V, as well as the interpeak latencies of wave I-III, wave III-V, and wave I-V, were notably longer in the severe NH group in contrast with those in the mild-to-moderate NH group (*p* < 0.05). Similarly, at a low stimulation rate of 51.1 times/s, the latency of waves I, III, and V, alongside the interpeak latencies of wave I-III and wave I-V, were also notably longer in the severe NH group (*p* < 0.05). These differences in interpeak latency of wave I-V (△IPL I-V) were consistently greater in the severe NH group across both stimulation rates of 11.1 times/s and 51.1 times/s (*p* < 0.05).

**Table 3:** Analysis of ABR results at high and low stimulation rates in severe NH group and mild-to-moderate NH group at 3 months of age (ms).

Groups	Stimulation rate	Latentperiod			Interpeak latency		51.1 times per second and 11.1 times per second Inter-interval differences of waves I-V	
		I	III	V	I-III	III-V	I-V	△IPL I-V
Severe NH group	11.1 times/s	1.70 ± 0.20	4.55 ± 0.19	6.48 ± 0.13	2.85 ± 0.29	1.93 ± 0.23	4.78 ± 0.22	-
Mild-to-moderate NH group	11.1 times/s	1.56 ± 0.20	4.25 ± 0.18	6.34 ± 0.20	2.70 ± 0.29	2.09 ± 0.27	4.79 ± 0.29	-
	*t*	3.82	8.36	4.44	2.78	−3.28	−0.03	
	*p*	<0.05	<0.05	<0.05	0.01	0.05	0.98	
Severe NH group	51.1 times/s	1.80 ± 0.24	4.65 ± 0.20	6.86 ± 0.19	2.85 ± 0.31	2.21 ± 0.23	5.06 ± 0.33	0.28 ± 0.39
Mild-to-moderate NH group	51.1 times/s	1.58 ± 0.21	4.31 ± 0.23	6.49 ± 0.22	2.73 ± 0.29	2.19 ± 0.30	4.92 ± 0.26	0.13 ± 0.38
	*t*	5.20	8.49	9.49	2.17	0.44	2.59	2.03
	*p*	<0.05	<0.05	<0.05	0.032	0.66	0.01	0.04

### 3.4 Comparative analysis of ABR outcomes at 6 months of age using high and low stimulation rates

The inter-group comparison of ABR results at 6 months of age, conducted at both high and low stimulation rates between the severe NH group and the mild-to-moderate NH group, is detailed in Table 4. At a low stimulation rate of 11.1 times/s, the latencies of waves I, III, and V, as well as the inter-wave intervals of I-III, III-V, and I-V, were notably longer in the severe NH group compared to the mild-to-moderate NH group (*p* < 0.05). This pattern was consistent at a higher stimulation rate of 51.1 times/s, where the severe NH group again displayed longer latencies for waves I, III, and V, and inter-wave intervals of I-III, III-V, and I-V (*p* < 0.05). At both stimulation rates of 11.1 times/s and 51.1 times/s, the difference in the inter-wave interval of I-V (△IPL I-V) was also notably greater in the severe NH group than in the mild-to-moderate NH group (*p* < 0.05).

**Table 4:** Analysis of ABR results at 6 months of age with high and low stimulation rates in severe NH group and mild-to-moderate NH group (ms).

Groups	Stimulation rate	latentperiod			Interpeak latency			51.1 times per second and 11.1 times per second Inter-interval differences of waves I-V
		I	III	V	I-III	III-V	I-V	△IPL I-V
Severe NH group	11.1 times/s	1.66 ± 0.21	4.51 ± 0.23	6.38 ± 0.31	2.86 ± 0.29	1.87 ± 0.42	4.73 ± 0.34	-
Mild-to-moderate NH group	11.1 times/s	1.52 ± 0.18	3.90 ± 0.21	6.01 ± 0.18	2.38 ± 0.28	2.11 ± 0.28	4.49 ± 0.25	-
	*t*	3.92	14.77	7.75	8.71	−3.57	4.13	
	*p*	<0.05	<0.05	<0.05	<0.05	0.05	<0.05	
Severe NH group	51. times/s	1.79 ± 0.20	4.68 ± 0.16	6.78 ± 0.19	2.89 ± 0.27	2.10 ± 0.24	4.99 ± 0.24	0.27 ± 0.35
Mild-to-moderate NH group	51.1 times/s	1.55 ± 0.15	4.21 ± 0.15	6.15 ± 0.31	2.66 ± 0.22	1.93 ± 0.36	4.60 ± 0.30	0.1 ± 0.27
	*t*	7.09	15.56	13.13	4.95	2.96	7.72	2.83
	*p*	<0.05	<0.05	<0.05	<0.05	0.01	<0.05	0.01

### 3.5 Intra-group comparisons of high-stimulation-rate ABR in neonates with severe and mild-to-moderate NH group at 3 and 6 months of age

This study conducted intra-group comparisons of high-stimulation-rate ABR among neonates diagnosed with severe and mild-to-moderate NH during their hospital stay, and subsequently at 3 and 6 months of age (Table 5). The findings are summarized as follows: (1) For the severe NH group, at a stimulation rate of 11.1 stimuli per second, the latency of wave V exhibited a significant reduction from the time of hospitalization, continuing through to 3 months and further to 6 months of age (*p* < 0.05). The interpeak latencies between waves III and V, as well as between waves I and V, were notably shorter at both 3 and 6 months than those recorded during hospitalization (*p* < 0.05). However, no significant changes were observed between the measurements at 3 and 6 months (*p* > 0.05). At a higher stimulation rate of 51.1 stimuli per second, similar patterns were observed: both the latency of wave V and the interpeak latency between waves III and V decreased progressively from hospitalization through to 3 and 6 months of age (*p* < 0.05). Additionally, the latency for wave III and the interpeak latency between waves I and V also showed reductions at 3 and 6 months compared to during hospitalization (*p* < 0.05), with no significant change noted between the 3-month and 6-month assessments (*p* > 0.05). At both stimulation rates of 11.1 and 51.1 stimuli per second, the difference in interpeak latency of waves I to V (△IPL I-V) was notably shorter at 3 and 6 months than during hospitalization (*p* < 0.05), but remained constant between the 3-month and 6-month evaluations (*p* > 0.05).

**Table 5:** Comparison of high-stimulation-rate ABR results in NH neonates aged 0–6 months (ms).

Groups	Stimulation rate	Latentperiod			Interpeak latency			51.1 times per second and 11.1 times per second Inter-interval differences of waves I-V
		I	III	V	I-III	III-V	I-V	△IPL I-V
Severe NH group 11.1 times/s	During hospitalization	1.72 ± 0.25	4.57 ± 0.20	6.66 ± 0.18	2.84 ± 0.29	2.10 ± 0.26	4.94 ± 0.32	–
	Three months old	1.70 ± 0.20	4.55 ± 0.19	6.48 ± 0.13^*^	2.85 ± 0.29	1.93 ± 0.23^*^	4.78 ± 0.22^*^	–
	Six months old	1.66 ± 0.21	4.51 ± 0.23	6.38 ± 0.31^*#^	2.86 ± 0.29	1.87 ± 0.42^*^	4.73 ± 0.34^*^	–
	*t*	1.30	0.83	22.29	0.02	7.44	7.93	–
	*p*	0.28	0.44	<0.05	0.98	0.05	0.05	–
51.1 times/s	During hospitalization	1.83 ± 0.25	4.80 ± 0.17	7.13 ± 0.18	2.97 ± 0.32	2.33 ± 0.25	5.30 ± 0.33	0.36 ± 0.42
	Three months old	1.80 ± 0.24	4.65 ± 0.20^*^	6.86 ± 0.19^*^	2.85 ± 0.31	2.21 ± 0.23^*^	5.06 ± 0.33^*^	0.28 ± 0.39^*^
	Six months old	1.79 ± 0.20	4.68 ± 0.16^*^	6.78 ± 0.19^*#^	2.89 ± 0.27	2.10 ± 0.24^*#^	4.99 ± 0.24^*^	0.27 ± 0.35^*^
	*t*	0.43	11.11	58.13	1.97	13.44	14.68	2.68
	*p*	0.65	<0.05	<0.05	0.14	<0.05	<0.05	0.04
Mild-to-moderate NH group 11.1 times/s	During hospitalization	1.65 ± 0.23	4.53 ± 0.21	6.62 ± 0.19	2.89 ± 0.34	2.08 ± 0.29	4.97 ± 0.28	–
	Three months old	1.56 ± 0.20^*^	4.25 ± 0.18^*^	6.34 ± 0.20^*^	2.70 ± 0.29^*^	2.09 ± 0.27	4.79 ± 0.29^*^	–
	Six months old	1.52 ± 0.18^*^	3.90 ± 0.21^*#^	6.01 ± 0.18^*#^	2.38 ± 0.28^*#^	2.11 ± 0.28	4.49 ± 0.25^*#^	–
	*t*	5.73	132.29	141.73	38.44	0.15	41.50	–
	*p*	0.01	<0.05	<0.05	<0.05	0.86	<0.05	–
51.1 times/s	During hospitalization	1.78 ± 0.21	4.67 ± 0.20	6.93 ± 0.24	2.89 ± 0.24	2.25 ± 0.31	5.14 ± 0.32	0.17 ± 0.40
	Three months old	1.58 ± 0.21^*^	4.31 ± 0.23^*^	6.49 ± 0.22^*^	2.73 ± 0.29^*^	2.19 ± 0.30	4.92 ± 0.26^*^	0.13 ± 0.38
	Six months old	1.55 ± 0.15^*^	4.21 ± 0.15^*#^	6.15 ± 0.31^*#^	2.66 ± 0.22^*^	1.93 ± 0.36^*#^	4.60 ± 0.30^*#^	0.10 ± 0.27
	*t*	20.05	80.29	119.35	10.79	14.45	46.31	0.56
	*p*	<0.05	<0.05	<0.05	<0.05	<0.05	<0.05	0.57

*p < 0.05 vs. during hospitalization; #*p* < 0.05 vs. 3 months of age.

(2) In the mild-to-moderate NH group, the following observations were recorded. At a low stimulation rate of 11.1 stimuli per second, both the latency of waves III and V and the interpeak latency of waves I-III and I-V showed a consistent decrease from the time of hospitalization to 3 months, and then further to 6 months of age (*p* < 0.05). The latency of wave I was shorter at both 3 and 6 months of age compared to during hospitalization (*p* < 0.05), with no significant differences observed between the 3-month and 6-month time points (*p* > 0.05). At a higher stimulation rate of 51.1 stimuli per second, similar patterns were observed: the latency of waves III and V, and the interpeak latency of waves III-V and I-V, progressively decreased from hospitalization to 3 months, and subsequently to 6 months of age (*p* < 0.05). Additionally, both the latency of wave I and the interpeak latency of waves I-III were reduced at 3 and 6 months compared to during hospitalization (*p* < 0.05), with no significant differences between the measurements at 3 and 6 months (*p* > 0.05). At both stimulation rates of 11.1 and 51.1 stimuli per second, the change in interpeak latency of waves I-V (△IPL I-V) in the mild-to-moderate NH group did not show any significant difference from hospitalization to 3 months and to 6 months of age (*p* > 0.05).

### 3.6 Waveform diagrams of ABR with high and low stimulation rates in children with high NH group

In the NH group, the ABR waveforms of the sixth child at both high and low stimulation rates during hospitalization, at 3 months old, and at 6 months old were randomly selected (Supplementary Figures 3A–C).

## 4 Discussion

### 4.1 Summary of findings

NH represents a significant risk factor for auditory dysfunction (13). In the ABR, the I wave originates from the cochlea/scala media, the II wave from the cochlear nucleus/scala media, the III wave from the superior olivary complex/cochlear nucleus, the IV wave from the lateral lemniscus/superior olivary complex, and the V wave from the inferior colliculus/medial geniculate body (14). Insufficient blood supply to brainstem structures such as the cochlear and vestibular nuclei in neonates with NH can compromise neuronal metabolism and lead to ischemia and edema within the auditory pathway. These pathological alterations may precipitate demyelination and conduction blocks in certain nerve fibers, thereby transforming the saltatory conduction of myelinated fibers into electrodiffusion and diminishing nerve impulse conduction velocity (15). ABR reflects synaptic potentials; as conduction velocity decreases, this results in prolonged latencies and interwave intervals at corresponding sites. The findings of the current study indicate that △IPLI-V was notably longer in the severe NH group compared to both the mild-to-moderate NH group and the healthy CG during hospitalization. Elevated levels of unconjugated and free bilirubin in the neonate’s serum can abnormally accumulate in critical brain regions, potentially leading to encephalopathy and adversely affecting various neural functions (16). Prior research has established that the auditory system is particularly susceptible to elevated bilirubin levels, predominantly impacting the auditory pathway and specifically the post-cochlear structures such as the brainstem auditory nuclei, inferior colliculus, spiral ganglion, and auditory nerve fibers. Without timely intervention, the auditory damage induced by NH may result in permanent impairment (17). Importantly, this study demonstrated that peak TSB levels were notably positively correlated with △IPLI-V in the severe neonatal NH group; this correlation was not observed in the mild-to-moderate NH group or the CG. Thus, it appears that TSB must exceed a certain threshold to exert a detectable impact on the auditory pathway.

Soni et al. observed that during a three-month follow-up of neonates diagnosed with simple NH, 61.61% exhibited reversible abnormalities in ABR (18). The present study reveals that at 3 months, △IPLI-V in neonates with severe neonatal NH was markedly higher than in those with mild-to-moderate NH, although there was improvement from in-hospital measurements; values nevertheless remained above the normal reference range. This persistence may be attributed to the enduring impact of bilirubin neurotoxicity on specific nuclei within the auditory pathway, particularly the superior olivary complex (17). At 6 months, △IPLI-V in the severe NH group remained notably higher than in the mild-to-moderate NH group, with no significant improvement from 3 months; waveform analysis indicated a predominant prolongation of the III-V interpeak interval. Zeng et al. reported that the length of time before treatment during which neonates experienced elevated serum bilirubin did not influence hearing outcomes. However, severe NH was found to affect hearing and preschool development in these children (19). Through longitudinal comparisons at hospitalization, 3 months, and 6 months, this study elucidated the dynamic alterations in ABR parameters among children with NH: recovery in the severe NH cohort was mainly observed from hospitalization to 3 months, with negligible improvements thereafter. This pattern may reflect the temporal disparities in neural repair mechanisms, specifically the early restoration of synaptic function followed by the more prolonged process of myelin regeneration (20).

### 4.2 Comparison with literature

Recent advancements in medical research have seen widespread application of ABR in the investigation of hearing impairments attributable to NH. For instance, Yadav et al. reported that the latencies of waves I, III, and V in ABR were extended in NH neonates, with 70–80% of these neonates exhibiting abnormal ABR results even after conventional discharge following bilirubin treatment (21). Conversely, another study on neonatal hearing screening in cases of NH found that serum bilirubin levels did not influence the outcomes of neonatal hearing screening (22). The current data extend these observations by demonstrating a positive correlation between peak TSB and △IPLI-V specifically in severe NH, and by showing that △IPLI-V at 3 months, while improved from in-hospital values, still exceeded normative ranges-aligning with Soni et al.’s report of reversible ABR abnormalities in 61.61% of cases (18), yet underscoring that reversal may be incomplete in a subset.

### 4.3 Explanation of mechanisms

The auditory brainstem pathology in NH-including ischemia, edema, demyelination, and conduction block -can shift fast, saltatory conduction toward slower electrodiffusion, increasing ABR latencies and interwave intervals (15). ABR reflects synaptic potentials; thus, as axonal conduction velocity falls, latencies at corresponding generators lengthen. Bilirubin is known to inhibit voltage-dependent calcium channels in the presynaptic membrane and disrupt neurotransmitter release, thereby delaying synaptic transmission (23); this effect becomes more pronounced under conditions of high stimulation rates, producing a greater I–V interpeak interval difference. Ye et al. observed that in an animal model of NH, even when ABR was severely abnormal or absent, there were no significant changes in the morphology or number of hair cells. However, there was a significant reduction in the density of spiral ganglion cells, accompanied by shrinkage of cell bodies, loss of axons, demyelination of cochlear auditory nerve fibers, and damage to type I afferent nerve endings (24). Morphological evidence is consistent with a primary impact on post-cochlear, brainstem auditory structures (17). The observed TSB-△IPLI-V correlation in severe NH likely reflects the extent of bilirubin-induced neuropathology required to measurably affect the auditory pathway, whereas the absence of such correlation in milder disease may indicate subthreshold effects. The predominance of III–V prolongation at 6 months further suggests a postsynaptic component, potentially indicating long-term alterations in glutamate receptor sensitivity (25).

### 4.4 Clinical implications

The persistence of △IPLI-V above the normal range at 3 months, despite improvement from in-hospital levels, indicates that a substantial proportion of severe NH cases may have residual auditory pathway dysfunction and could benefit from ongoing audiologic surveillance. Given the greater sensitivity of high-stimulation-rate ABR to postsynaptic abnormalities, testing at 3 months may help identify high-risk neonates who would benefit from early intervention. The lack of further improvement and the specific III–V interval prolongation at 6 months may signal long-term or potentially permanent neural damage (17, 25); accordingly, high-stimulation-rate ABR at 6 months is important for prognosis and for planning rehabilitation strategies. Collectively, these findings support longitudinal ABR monitoring in NH, with particular attention to the three-month recovery phase as a key prognostic checkpoint.

## 5 Limitations and future directions

This study methodically assessed the utility of high-stimulation-rate ABR for monitoring auditory function in children with NH. In comparison to standard ABR, high-stimulation-rate ABR exhibits increased sensitivity to synaptic dysfunction, thereby enabling earlier and more precise detection of neural impairment. Our findings advocate for the integration of high-stimulation-rate ABR into the routine follow-up regimen for children with severe NH. Nonetheless, the study is not without limitations. Firstly, while our study design included a healthy control group for baseline comparisons during the hospitalization phase, we acknowledge the significant limitation of not having longitudinal ABR data for this control group at 3 and 6 months. This absence impacts the interpretation of our findings in two important ways: (1) Impact on ΔIPL Threshold Interpretation: Without age-matched normative controls, we cannot establish definitive ΔIPL thresholds for normal auditory maturation during the critical 0–6 month period. The ‘partial recovery’ observed in severe NH neonates (ΔIPL I-V shortening from hospitalization to 3 months) is interpreted relative to the mild–moderate group rather than against normal developmental benchmarks. This limits our ability to determine whether the persistent abnormalities in severe cases represent delayed maturation or permanent injury. (2) Impact on Developmental Effect Assessment: The missing control data prevents direct quantification of how hyperbilirubinemia affects the normal trajectory of auditory pathway maturation. While our within-group comparisons show significant changes over time, we cannot distinguish whether the stability observed in mild–moderate cases reflects normal development or pathological stabilization. Future studies should include longitudinal control monitoring to establish normal ΔIPL maturation patterns and better quantify the developmental impact of bilirubin neurotoxicity. Secondly, the collection of high-stimulation-rate ABR data was confined to the first 6 months post-birth, with no data available for extended follow-up periods. Subsequent studies should focus on longitudinal assessments to better understand the developmental trajectories of the auditory system in children affected by NH. The absence of ROC analysis limits the establishment of clinical diagnostic thresholds. Future studies with larger samples will incorporate ROC analysis to define optimal cut-off values.

## 6 Conclusion

High-stimulation-rate ABR has been proven to effectively ascertain auditory impairments in children with NH. Children with severe NH exhibit persistent auditory abnormalities, whereas those with mild-to-moderate NH demonstrate only transient alterations. This distinction underpins a reliable methodology for the early identification of children at elevated risk, supporting the inclusion of high-stimulation-rate ABR in routine clinical follow-up protocols for children with significant NH levels.

## Data Availability

The original contributions presented in the study are included in the article/Supplementary material, further inquiries can be directed to the corresponding author/s.
