# Supplementary material for: High-stimulation-rate ABR predicts persistent auditory pathway dysfunction in neonates with severe hyperbilirubinemia: a 6-month longitudinal study
Source: Front Neurol. 2026 Jan 12;16:1687189. doi: 10.3389/fneur.2025.1687189 (PMC12833519; doi:10.3389/fneur.2025.1687189)
Supplement: Supplementary file 1 [file Table_1.docx]

Neonates from the Longquanyi District Maternal and Child Health Hospital in Chengdu, spanning from January 2022 to January 2025. All the subjects met the inclusion and exclusion criteria.

During the hospitalization period, high- and low-stimulation-rate ABR, TEOAE, and acoustic impedance tests were performed.

The latency values of waves I, III and V, the inter-wave intervals, and the difference in inter-wave intervals ΔIPLⅠ-Ⅴ between waves I and V were analyzed under high (51.1 times/s) and low (11.1 times/s) stimulation rates.

56 cases of NH children (112 ears in total): severe NH group and mild-to-moderate NH group

Healthy CG: 28 newborns (56 ears)

During the hospitalization period, high-stimulation-rate ABR, TEOAE, and acoustic impedance tests were performed.

At 3 months of age, high- and low-stimulation-rate ABR, TEOAE, and acoustic impedance tests were performed.

At 6 months of age, high- and low-stimulation-rate ABR, TEOAE, and acoustic impedance tests were performed.

Supplementary Figures 1 The test flowchart .

| 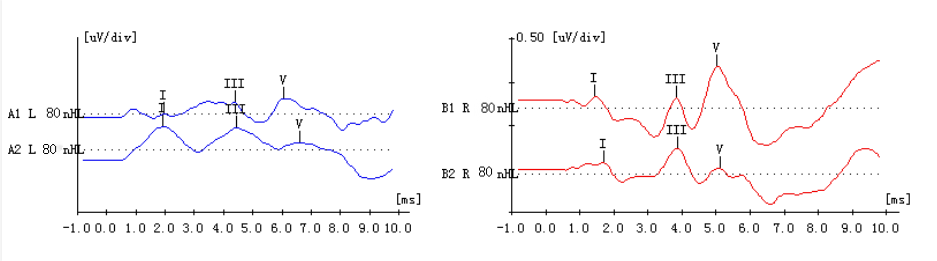 |
| --- |
| Figure 3A ABR of NH children during hospitalization at low and high stimulation rates: A1 L: ABR of the left ear at a low stimulation rate (11.1 times/s), A2 L: ABR of the left ear at a high stimulation rate (51.1 times/s); B1 R: ABR of the right ear at a low stimulation rate (11.1 times/s), B2 R: ABR of the right ear at a high stimulation rate (51.1 times/s). |
| 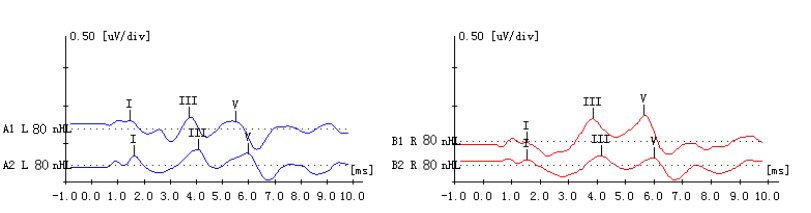 |
| Figure 3B ABR of NH neonates at 3 months of age with low and high stimulation rates: ABR of NH neonates during hospitalization with low and high stimulation rates: A1 L: ABR of the left ear with a low stimulation rate (11.1 times/s), A2 L: ABR of the left ear with a high stimulation rate (51.1 times/s); B1 R: ABR of the right ear with a low stimulation rate (11.1 times/s), B2 R: ABR of the right ear with a high stimulation rate (51.1 times/s). |
| 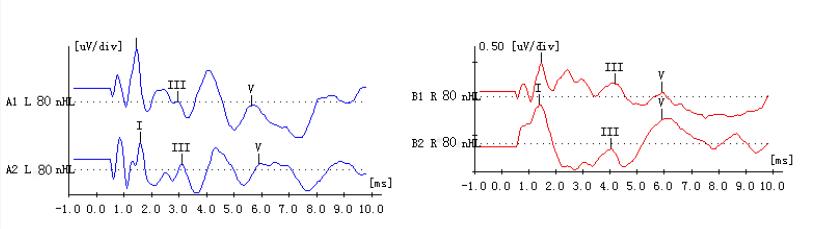 |
| Figure 3C ABRs of CNH neonates at 6 months of age with low and high stimulation rates: ABRs of NH neonates at 3 months of age with low and high stimulation rates: ABRs of NH neonates during hospitalization with low and high stimulation rates: A1 L: ABR of the left ear at a low stimulation rate (11.1 times/s); A2 L: ABR of the left ear at a high stimulation rate (51.1 times/s); B1 R: ABR of the right ear at a low stimulation rate (11.1 times/s); B2 R: ABR of the right ear at a high stimulation rate (51.1 times/s). |

Supplementary Figures 3 A-C
